# An Experimental Verification of the Predicted Effects of Promoter TATA-Box Polymorphisms Associated with Human Diseases on Interactions between the TATA Boxes and TATA-Binding Protein

**DOI:** 10.1371/journal.pone.0054626

**Published:** 2013-02-12

**Authors:** Ludmila Savinkova, Irina Drachkova, Tatyana Arshinova, Petr Ponomarenko, Mikhail Ponomarenko, Nikolay Kolchanov

**Affiliations:** 1 Institute of Cytology and Genetics, Siberian Division, Russian Academy of Sciences, Novosibirsk, Russia; 2 Novosibirsk State University, Novosibirsk, Russia; Consejo Superior de Investigaciones Cientificas, Spain

## Abstract

Human genome sequencing has resulted in a great body of data, including a stunningly large number of single nucleotide polymorphisms (SNPs) with unknown phenotypic manifestations. Identification and comprehensive analysis of regulatory SNPs in human gene promoters will help quantify the effects of these SNPs on human health. Based on our experimental and computer-aided study of SNPs in TATA boxes and the use of literature data, we have derived an equation for TBP/TATA equilibrium binding in three successive steps: TATA-binding protein (TBP) sliding along DNA due to their nonspecific affinity for each other ↔ recognition of the TATA box ↔ stabilization of the TBP/TATA complex. Using this equation, we have analyzed TATA boxes containing SNPs associated with human diseases and made *in silico* predictions of changes in TBP/TATA affinity. An electrophoretic mobility shift assay (EMSA)-based experimental study performed under the most standardized conditions demonstrates that the experimentally measured values are highly correlated with the predicted values: the coefficient of linear correlation, r, was 0.822 at a significance level of α<10^−7^ for equilibrium *K*_D_ values, (-ln *K*_D_), and 0.785 at a significance level of α<10^−3^ for changes in equilibrium *K*_D_ (δ) due to SNPs in the TATA boxes (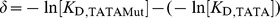). It has been demonstrated that the SNPs associated with increased risk of human diseases such as α-, β- and δ-thalassemia, myocardial infarction and thrombophlebitis, changes in immune response, amyotrophic lateral sclerosis, lung cancer and hemophilia B Leyden cause 2–4-fold changes in TBP/TATA affinity in most cases. The results obtained strongly suggest that the TBP/TATA equilibrium binding equation derived can be used for analysis of TATA-box sequences and identification of SNPs with a potential of being functionally important.

## Introduction

Single nucleotide polymorphisms (SNPs) represent the commonest type of genetic variation in man. Most of more than 15 million annotated polymorphisms are located in DNA coding regions, which makes the mechanism of their action on the phenotype perfectly clear: a certain protein will be deficient [1]. Polymorphisms in regulatory regions and the effects of these polymorphisms on gene expression remain to be much less well studied. Many of non-coding SNPs lie within regulatory DNA motifs, altering their affinity for transcription factors and thus also altering the expression levels of genes cis-targeted by those motifs, which accounts for differences in responses to external and internal signals, propensity to certain diseases, and sensitivity to therapy, to mention a few. Thus, information on polymorphisms represents a huge resource for biomedical studies, diagnostics and drug development.

The transcription of any protein-coding gene by RNA polymerase II starts with assembly of the basal transcriptional machinery on the core promoter. The core promoter is the term for the DNA region spanning ∼100 nucleotides to the left (in the 5′-region) and to the right (in the 3′-region) from the transcription start site, with a variable number of regulatory regions [2] such as the TATA box, BRE (TFIIB recognition element), Inr (initiator element), MTE (motif ten element), DPE (downstream promoter element), DCE (downstream core element), and XCPE1 (X core promoter element 1) [3] and others.

The TATA box is the most well-studied core promoter element, even though the TATA-containing promoters make up only 10–16% of the genes read by RNA polymerase II, of which only 30% contain the canonical TATA box, which provides evidence that the canonical TATA-box sequence, TATAAAA, is variable in natural promoters [4]. The formation of the basal transcription complex on the TATA-containing promoters of genes read by RNA polymerase II starts with the recognition and binding of the TATA box by the TATA-binding protein (TPB), a subunit of the transcription factor TFIID.

It has been demonstrated that identical TATA-box mutations in different promoters have different effects on promoter activity [5]. Although TATA boxes are so much variable, some TATA-containing promoters can be very sensitive to mutations in the TATA boxes. This statement is supported by molecular-genetically and clinically identified single nucleotide polymorphisms associated with increased risk of hereditary diseases and found in the TATA boxes of human gene promoters. [6]. The importance of knowing more about SNPs in regulatory regions (in particular, the TATA box) is even more relevant because such SNPs make individual sensitivity to bacterial and viral infection as well as the therapeutic drugs used for treatment individual. It is especially important to know how to identify and study the SNPs that are associated with risk of widespread polygenic (complex) human diseases such as arthritis, hypertension, cancer, Alzheimer disease and others.

Obviously, no search for functionally importance SNPs, their analysis or systematization are possible without the use of bioinformatic approaches. However, many of the approaches that have been developed to date are not used for the lack experimental verification. To fill in the gap, we are currently running a comprehensive experimental and computer-aided study of SNPs in TATA boxes and their effects on the interactions between TPB and TATA boxes. Based on our earlier experimental [7] and computer-aided study of TBP/TATA interactions and the literature data, we had previously developed a model [8] describing the binding of TPB to TATA boxes in three successive steps: TPB sliding along DNA [4], [9] due to their nonspecific affinity for each other [7], [8] ↔ recognition of the TATA box [10] ↔ stabilization of the TBP/TATA complex [11]. With the use of that model, we have analyzed interactions between TPB and TATA boxes in human gene promoters containing SNPs associated with diseases and also determined *in silico* changes in the affinity of TPB for those SNPs. In the current work, we have performed, under the most standardized experimental conditions, a study of interactions between recombinant human TBP (hTPB) and nucleotides identical to the TATA boxes in the promoters of the genes for α, β, and εψ globins, tissue factor, triosephosphate isomerase, NO synthase, superoxide dismutase, cytochrome P450 2A6, mannose-binding lectin, coagulation factor IX and interleukin-1 beta without and with SNPs associated with various pathologies. The obtained equilibrium *K_D_* values, which characterize TBP/TATA affinity without and with SNPs in TATA boxes, are highly correlated with predicted values.

## Results and Discussion

The table 1 presents the experimentally obtained values of equilibrium dissociation constants (*K_D_*), which characterize the affinity of hTPB for ODNs. The ODNs are identical to TATA boxes with the flanking regions of the promoters of the genes being studied in healthy individuals and patients with SNPs in the TATA boxes in the promoters of the genes being studied. Also, the table 1 presents negative natural logarithms of the predicted and experimentally determined values of *K_D_* and changes in *K*_D_ (δ) when TATA boxes contained SNPs: 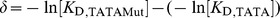.

**Table 1:** The experimental verification of predicted changes in *K*_D_ for TBP/TATA when the TATA boxes contain SNPs associated with human diseases.

Gene	Sequences (5′ –3′ strands)	Allele	Norm/disease association	Prediction		Experiment		
				-ln[*K*_D_]	δ	*K*_D_, nM	-ln[*K*_D_]	δ
*Hbβ*	cagggctgggCATAAAAgtcagggca	WT	Norm [16]	18.47		44±3	16.94	
	cagggctgggCGTAAAAgtcagggca	−31A>G	β-thalassemia [18]	17.87	−0.60	99±9	16.13	−0.81
	cagggctgggCAAAAAAgtcagggca	−30T>A	β-thalassemia [16]	17.01	−1.46	116±5	15.97	−0.97
	cagggctgggCACAAAAgtcagggca	−30T>C	β-thalassemia [14]	17.49	−0.98	111±8	16.01	−0.93
	cagggctgggCATGAAAgtcagggca	−29A>G	β-thalassemia [12]	17.23	−1.24	390±20	14.76	−2.18
	cagggctgggCATAGAAgtcagggca	−28A>G	β-thalassemia [15]	17.76	−0.71	87±8	16.26	−0.68
	cagggctgggCATACAAgtcagggca	−28A>C	β-thalassemia [17]	17.94	−0.53	300±20	15.02	−1.92
	cagggctgggCATAATAgtcagggca	−27A>T	β-thalassemia [13]	18.11	−0.36	64±3	16.56	−0.38
*Hbδ*	acaggaccagCATAAAAggcagggca	WT	Norm [19]	18.94		46±5	16.89	
	acaggaccagCGTAAAAggcagggca	−31A>G	δ-thalassemia [19]	18.33	−0.61	120±10	15.94	−0.95
*Hbξψ*	ctgccacacccaCATTATTagaaaat	WT	Norm [20]	17.72		152±6	15.70	
	ctgccacaccCACATTATCagaaaat	−70T>C	α-thalassemia [20]	18.28	+0.56	112±6	16.00	+0.30
*CYP2A6*	tttcaggcagTATAAAggcaaaccac	WT	Norm [28]	19.87		17±4	17.89	
	tttcaggcagTAGAAAggcaaaccac	−48T>G	Lung cancer [28]	18.38	−1.49	80±20	16.34	−1.55
*SOD1*	aggtctggccTATAAAgtagtcgcgg	WT	Norm [31]	19.21		40±8	17.03	
	aggtctggccTGTAAAgtagtcgcgg	−27A>G	Amyotrophic lateral sclerosis [31]	18.04	−1.17	170±30	15.59	−1.44
*MBL2*	catctatttcTATATAgcctgcaccc	WT	Norm [37]	19.68		28±4	17.39	
	catctatttcTACATAgcctgcaccc	−35T>C	Immunosupression [37]	18.57	−1.11	58±9	16.66	−0.73
*TPI*	cgcggcgctcTATATAAgtgggcagt	WT	Norm [32]	20.11		4.8±0.7	19.15	
	cgcggcgctcTATAGAAgtgggcagt	−24T>G	Neurological and muscular disorders [32]	19.08	−1.03	150±20	15.71	−3.41
*FIX*	acagctcagcTTGTACTTTggtacaa	WT	Norm [39]	18.24		510±60	14.49	
	acagctcagcTTCTACTTTggtacaa	−26G>C	hemophilia B Leyden [39]	17.75	−0.49	500±100	14.51	0.02
*IL1B*	ttttgaaagcCATAAAAAcagcgagg	WT	Norm [29]	18.67		29**±**4	17.36	
	ttttgaaagcTATAAAAAcagcgagg	−31C>T	lung cancer [29]	19.85	+1.18	7**±**1	18.78	+1.42
*TF*	gccggcccTTTATAgcgcgcggggca	WT	Norm [41]	18.91		72±9	16.45	
	gccggcccTTTATAgTgcgcggggca	−21C>T	thrombophlebitis and myocardial infarction [41]	19.43	+0.52	26±3	17.47	+1.02
*NOS2A*	atggggtgagTATAAATActtcttgg	WT	Norm [38]	19.85		1.8±0.2	20.14	
	atggggtgagTATAAATAcCtcttgg	−21T>C	resistance to infections [38]	20.06	+0.21	1.6±0.3	20.25	+0.11

*K*_D_± (standard deviation); δ - the difference between the affinity of hTBP for ODNs with and without SNPs in their TATA boxes expressed as natural logarithms, 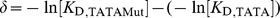.

### SNPs in the TATA Boxes of the *β*-, *δ*- and εψ-globin Genes

It has been demonstrated [12]–[20] that SNPs in the TATA boxes of the promoters of the β-, δ-globin genes and the εψ-globin pseudogene (*HbB*, *HbD*, and *Hb εψ*, respectively) in man lead to β-, δ- and α-thalassemias of varying severity due to disruption of the balance in the synthesis of structurally normal globin chains, which compose normal hemoglobins: A (α_2_β_2_, the commonest structural unit of hemoglobin in adult humans, HbA, with an amount of ∼97%) and A2 (α_2_δ_2_, with an amount of ∼3%) [21]. Most commonly affected is the synthesis of α- and β-globin chains, which corresponds to α- and β-thalassemia, respectively. Imbalances of any of three globin chains cause their aggregation, hemolysis and failure in erythropoiesis. The forms of hemoglobinopathies are mild, moderate and severe. While patients with mild and moderate hemoglobinopathies may have asymptomatic anemia and a normal quality of life, patients with severe hemoglobinopathies may have serious disorders such as hemolytic anemia, skeletal abnormalities, poor growth, jaundice, be dependent on transfusions, to name a few [22]. δ-thalassemia has a lower prevalence because in healthy adult humans hemoglobin A2 (α2δ2), which consists of two δ-globin chains, exists in an amount of ∼3% of hemoglobin A (α2β2) [19].

As can be seen from the sequences presented in the table 1, the first T in the TATA box of the β- and δ-globin genes in a healthy individual is replaced by C: the TATA box appears as CATAAAA instead of canonical TATAAAA [16]. The effects of this substitution in the TATA box have been studied by a range of researchers. The use of TPB from the yeast *S. cerevisiae* [23] demonstrated that this substitution has little effect on binding. It has also been demonstrated [24] that the promoter containing the CATAAAA sequence is 1/40 as efficient in inducing transcription *in vivo* as the classical TATAAAA sequence. It has also been demonstrated [25] that transcription in HeLa is decreased three-fold when the first T in the TATA box is replaced by C (TATAAAA→CATAAAA). As can be seen from these examples, a T to C substitution at position 1 in the TATA box variously affect transcription and, therefore, TBP/TATA interactions. It is possible that these differences are due to differences in the sequences that flank the TATA box and differences in experimental conditions. It has been demonstrated [26], [7] that the affinity of TPB for oligonucleotides with different abundances of AT pairs in the sequences flanking the TATA box can be 25–30 times as different. Our estimate of the affinity of TBP for the TATA box in the β-globin gene of healthy individuals (WT in the table 1) is *K*_D_ = 44 nM.

Analysis of the equilibrium *K*_D_ values for the TATA boxes in the SNP-containing promoters of the β- and δ-globin genes demonstrates that a 2–2.6-fold decrease in affinity is associated with thalassemia intermedia or thalassemia minor. Affected individuals do not depend on red blood cell transfusions and normally have a good quality of life. Only the 28A>C mutation [17] found in two Kurdish individuals, brothers aged 1.5 years, is associated with thalassemia major: both patients were dependent on red blood cell transfusions. Analysis revealed partial or total lack of normal β-globin mRNAs in them. The use of a vector with a similar mutation (TATAAAA → TATACAA) in HeLa led to a 20-fold decrease in transcription as compared to normal levels [25], which is good agreement with the characteristic that we obtained for the interaction between hTPB and ODN containing this substitution: K_D_ = 300 nM, which is 1/8 as much as the normal affinity. The -29A>G SNP led to a nearly 9-fold decrease in hTBP/TATA affinity (*K*_D_ = 390 nM) and in that case β-thalassemia intermedia was detected in an individual with a practically normal quality of life. In a work devoted to artificial mutagenesis of the β-globin gene [27], the conclusion was made that 15–30% hTBP/TATA binding is enough for transcription at normal levels. Our quantitative estimates of TBP/TATA binding (see the table 1, β globins) and the mRNA amounts provided in some works [12]–[18] (*K*_D_ = 390 nM, 25% of mRNAs; *K*_D_ = 300 nM, no mRNAs; *K*_D_ = 87 nM, 10% of mRNAs; *K*_D_ = 64 nM, 20% of mRNAs, *K*_D_ = 116 nM, 8–13% of mRNAs; *K*_D_ = 99 nM, 50% of mRNAs) fail to make us reach the same conclusion and suggest that regulation of β-globin gene transcription *in vivo* and *in vitro* occurs on a more integrated and individual basis. We have determined the affinity of hTPB for the TATA boxes in the δ-glodin gene and the εψ-glodin pseudogene with SNPs associated with δ- and α-thalassemia intermedia. The affinity of hTPB to the TATA box is decreased 2.6-fold in δ-thalassemia and is increased 1.3-fold in α-thalassemia.

### SNPs in the TATA Boxes of the *CYP2A6* (Cytochrome P450) and *IL1B* (Interleukin-1 Beta) Genes

The products of these genes, nicotine oxidase and proinflammatory cytokine, play a role in carcinogen activation, drug detoxification and the formation of inflammatory cell responses.

In some patients with lung cancer, the -48T>G polymorphism was detected in the TATA box of the *CYP2A6* gene promoter [28], and in some, −31C>T in the TATA box of the *IL1B* gene promoter [29]. The −48T>G substitution destroys the TATA box in the gene encoding nicotine oxidase (*CYP2A6*) [28] and is associated with increased risk of inflammatory diseases and lung cancer in smokers: the hTBP/TATA affinity is decreased 4.7-fold. When the −31C>T polymorphism in the TATA box of the *IL1B* gene was the case, we demonstrated that the hTBP/TATA affinity was increased more than fourfold: *K*_D_ = 29 nM in healthy people and *K*_D_ = 7 nM in affected people. This increase is because the TATA-box sequence become a consensus sequence and is associated with increased risk of inflammatory diseases and non-small-cell lung carcinoma in a cohort of Norwegian patients [29] and with risk of hepatocellular carcinoma in Japanese patients with chronic hepatitis C virus infection. [30].

### SNPs in the TATA Boxes of the *SOD1* (Superoxide Dismutase 1) and *TPI* (Triosephosphate Isomerase) Genes

TATA-box polymorphisms in these genes are associated with diseases that cause neurological and muscular disorders. Two patients with amyotrophic lateral sclerosis were reported [31] to have the −27A>G polymorphism in the TATA box (TGTAAA instead of TATAAA) of the *SOD1* gene encoding the enzyme superoxide dismutase 1. We have demonstrated a 4.2-fold decrease in hTBP/TATA affinity: *K*_D_ = 40 nM in healthy people and *K*_D_ = 170 nM in affected people. The *TPI* gene is a housekeeping gene. Triosephosphate isomerase, which is the enzyme that this gene encodes, is involved in glycolysis and occurs in every organism. If an SNP causes its deficiencies, neuromuscular disorders and hemolytic anemia are expected [32]. Additionally, it has recently been demonstrated [33] that triosephosphate isomerase in stomach cancer can convert drug-resistant cells into drug-sensitive cells, which renders chemotherapy more effective and makes this enzyme appear as a candidate target for new drugs against stomach cancer. It has been demonstrated that mutations causing deficiencies in TPI are associated with chronic hemolytic anemia, degenerative neurological disorders, cardiomyopathy, infant mortality and more [32]. With the −24T>G SNP in the TATA box of this gene, a very dramatic (more than 30-fold) decrease in TBP/TATA affinity was demonstrated; that decrease being correlated with a low gene expression level [32]. As can be seen from the case with the SNP-containing TATA box in the *TPI* gene promoter, even a very strong (41-fold) change in TBP/TATA affinity is associated with a decrease in gene expression and enzyme activity in erythrocytes: the TPI activity is decreased by 80–98% in some patients and by 26–50% *in vivo* in heterozygous individuals [34].

### SNPs in the TATA Boxes of the *MBL2* (Mannose-binding Lectin) and *NOS2A* (NO Synthase) Genes

The products of these genes, mannose-binding lectin and NO synthase, are involved in many responses produced by the organism, including the immune response. As is known, genetically determined variation in MBL concentrations in human blood serum accounts for varying sensitivity to infections and predisposition to autoimmune, metabolic and cardiovascular diseases [35]. Low MBL levels are associated with increased risk of recurrent infections [36]. We have demonstrated a twofold decrease in TBP/TATA affinity when the TATA box of this gene contains the −35T>C SNP [37]. A slight increase in TBP/TATA affinity caused by the −21T>C polymorphism [38] located near the TATA box in the promoter of the *NOS2A* gene encoding NO synthase is associated with increased resistance to diseases such as malaria, acute respiratory and lung diseases.

### SNPs in the TATA Boxes of the *FIX* and *TF* Genes

Polymorphisms in the coagulation factor IX and tissue factor gene promoters are associated with vascular diseases. The promoter of the wild-type clotting factor IX gene contains a region for binding to the hepatocyte nuclear factor HNF4 located in the TATA box. HNF4 is the main factor controlling coagulation factor IX expression in healthy individuals and when it is unable to effectively bind to an altered site, the individual will develop hemophilia B Leyden.

It has been found [39] that the −26G >C mutation reduces HNF4 binding to the background level. TPB binds to this site with a very low specificity: *K*_D_ is 510 nM without this polymorphism and 500 nM with this polymorphism.

TF is a transmembrane protein expressed in many tissues, including the outermost layer of the vessel walls, where it rapidly activates coagulation whenever integrity is compromised [40]. A 2.7-fold increase in affinity with −21C>T SNP in the TATA element of the tissue factor gene is consistent with the known enhancement in gene expression and increased risk of thrombophlebitis and myocardial infarction [41].

### Statistical Data Analysis

**Figure 1:**
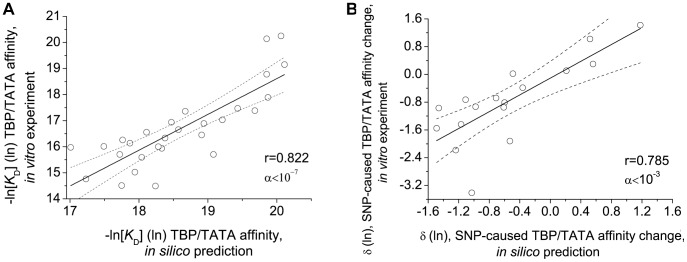
The experimentally measured affinity and affinity change are highly correlated with the predicted values. (**A**) The correlation between the TBP/TATA affinity as predicted *in silico* and measured *in vitro.* The coefficient of linear correlation, r, is 0.822 at a significance level of α <10^−7^. (**B**) The correlation between SNP-caused TBP/TATA affinity change (δ) as predicted *in silico* and measured *in vitro.* The coefficient of linear correlation, r, is 0.785 at a significance level of α<10^−3^. 95% confidence intervals for linear regression built using the package STATISTICA.

A comparison of the experimentally obtained and previously predicted values of changes to TBP/TATA affinity when the TATA boxes contained mutations was performed using the standard software program package STATISTICA [42] and demonstrated that these values were well correlated to each other. Fig. 1 presents the 95% confidence intervals for the linear regressions built. As can be seen, some of the absolute values as predicted for TBP/TATA affinity, *K*_D_, and mutational changes, δ, do not fall within the respective confidence intervals. We found no significant correlation between excursions outside the 95% confidence intervals and the controlled parameters that we used for *in silico* predictions and experimental measurements made *in vitro*. This implies that some binding parameters remain to be included in the equation for TBP/TATA binding in three successive steps that we are verifying. The observed *in silico* underprediction of TPB affinity and mutational changes in TATA boxes suggests that damage events in DNA sequence context can produce a cooperative effect at the TBP/TATA binding site. Indeed, the universal molecular processes that have an influence on TBP/TATA binding include the specific packaging of the core promoter into nucleosomes between positions −70 and +30 relative the transcription start site [43], with position −43 being the one of the nucleosome center [44]. The binding site of the nucleosome center on the DNA is AT-rich and matches with the optimal localization of TATA boxes [45]. This suggests that eukaryotic promoters are likely to possess the composite element \(H3-H4)(H2A-H2B) (H3-H4), which was detected experimentally [44], [45], but has not yet been considered for use in tools developed for in *in silico* analysis.

### Conclusions

As is known, completion of the Human Genome Project has resulted in a wealth of new data and posed new challenges. For example, a large number of SNPs with unknown phenotypic manifestations have been found. Consequently, identification and analysis of regulatory SNPs in human genes will help quantify the effects of these SNPs on human health and sensitivity to drugs and environmental factors.

Transcriptional regulation of gene expression is performed by a large number of proteins and protein complexes, which interact with DNA and one another and cooperatively stimulate or inhibit gene expression in response to internal and external signals. Transcription factors are the key players in this process. The interaction between TPB and the TATA box on TATA-containing promoters is one of the rate-limiting stages of transcription initiation. Relationships between the quantitative characteristics of TBP/promoter interactions and gene transcription levels are very important for understanding the mechanisms of activation and inhibition of gene transcription and expression; however, these data, especially on man, are desperately needed. The existing literature data on the interaction between TPB and the TATA box have been obtained using different TPBs: from *S. cerevisiae*, *D. melanogaster*, mice, man, full-length TBP molecules and its DNA-binding domain. The model systems used, too, were different (yeast, flies, laboratory animals, human cell lines, to name a few), and so were the experimental conditions, which prevents comparisons of the results so obtained or making inferences about the effect that a particular substitution in the TATA box has on TBP/TATA affinity. In the current work, we have performed, under the most standardized experimental conditions, a study of interactions between recombinant human TBP (a full-length molecule with the amino acid composition as in the natural human TBP molecule) and 28 ODN identical to the TATA boxes in gene promoters in healthy people and patients whose diseases are associated with SNPs in TATA boxes. As can be seen from the results provided herein, the wild-type natural promoters of four genes studied (the *β-*globin, *δ-*globin, *εψ-*globin and *IL1B* genes) contain CATA boxes instead of TATA boxes, three genes (*NOS2A, SOD1* and *CYP2A6*) contain classical TATA-box sequences, and two genes (*TPI* and *MBL*) contain TATATA boxes. The highest affinity (*K*_D_ = 1.8 nM and *K*_D_ = 4.8 nM) was observed for the TBP/TATA interaction in the *NOS2A* and *TPI* genes, in which the TATA-box sequences are classical, TATAAAA and TATATA, respectively. The lowest affinity (*K*_D_ = 72 nM) was observed for the *TF* gene, in which the TATA-box sequence has a reduced sequence (TTTATA) in healthy people. It has now been experimentally demonstrated for the first time that TBP/TATA affinity in monogenic and polygenic pathologies is not beyond the range between ¼ and 4 times the wild-type value on most occasions. As can be seen from the comparison of our results with the literature data, the human organism possesses large compensatory abilities [46] and that the same SNPs can have different effects on human health. The high values of the coefficients of linear correlation between the predicted and experimentally obtained results (-ln[*K*_D_] and δ) strongly suggest that the equation for TBP/TATA equilibrium binding we had previously derived is applicable for analysis of TATA-box sequences, prediction of changes in the affinity of TPB for SNP-containing TATA boxes and its experimental verification for identification of regulatory SNPs with potential functional importance and prediction of their effects on phenotypic traits.

## Materials and Methods

### Protein Expression and Putification

Recombinant full-length human TBP containing only the native amino acid sequence was overexpressed in *E. coli* BL21(DE3) cells transformed with pAR3038-hTBP (plasmid pAR3038-hTBP was the kind gift of Prof. B. Pugh, Pennsylvania State University). Expression of hTPB was as described by Pugh [47] with modifications (the IPTG concentration were 1 mM instead of 0.1 mM and the induction time was 3 h instead of 1.5 h). TBP was purified to homogeneity using three-step procedure involving polyethylenimine precipitation, phosphocellulose chromatography, and ammonium sulfate precipitation as described by Pugh [47]. Based on Coomassie Brilliant Blue R 250 stained SDS-PAGE analyses coupled with scanning densitometry, the purity of these TBP preparation was determined to be more than 98%. The total protein concentration was determined by Bradford [48]. The concentration of active TBP was determined by titrations of TBP against known concentrations of TATA AdML, which were well above the *K*_D_, and was about 50% from total protein concentration.

### Labeling Oligodeoxyribonucleotides with ^32^P

Twenty-six base pair oligodeoxyribonucleotides (ODNs) synthesized and additionally purified by electrophoresis in PAGE (Biosset, Novosibirsk) were used. The ODN sequences used were identical to the TATA boxes (with and without SNPs) with the flanking sequences of the β-globin gene, δ-globin gene, εψ-globin pseudogene, and the genes for tissue factor, triosephosphate isomerase, NO synthase 2A, superoxide dismutase 1, cytochrome P450 2A6, mannose-binding lectin, clotting factor IX and human interleukin-1 beta cited from works referenced in the table 1. ODNs quality was tested for with the use of MALDI TOF MS (Bruker Daltonics). Labeled double-stranded ODNs were obtained by labeling both strands with ^32^P-ATP (Biosan, Novosibirsk) using T4 polynucleotide kinase (SibEnzime, Novosibirsk), annealing at 95^o^ C (at equimolar concentration) and slowly (for not less than 3 h) cooling to room temperature. Duplexes were analyzed in 15% non-denaturing PAGE (1x TBE) [49], isolated and purified by electroelution.

### Measurements of the Equilibrium Dissociation Constants for hTBP/TATA Complexes

The equilibrium dissociation constants (*K*_D_) for the complexes of hTPB with TATA-containing double-stranded ODNs identical to wild-type and SNP-containing TATA-box variants were measured using a traditional approach, which included titration of a fixed amount of active TBP (typically 0.3 nM) with the increasing concentrations of TATA-containing ODN to reach equilibrium. The time to reach equilibrium was determined previously for each ODN. Each K_D_ value was determined following not less than 8 experimental runs.

**Figure 2:**
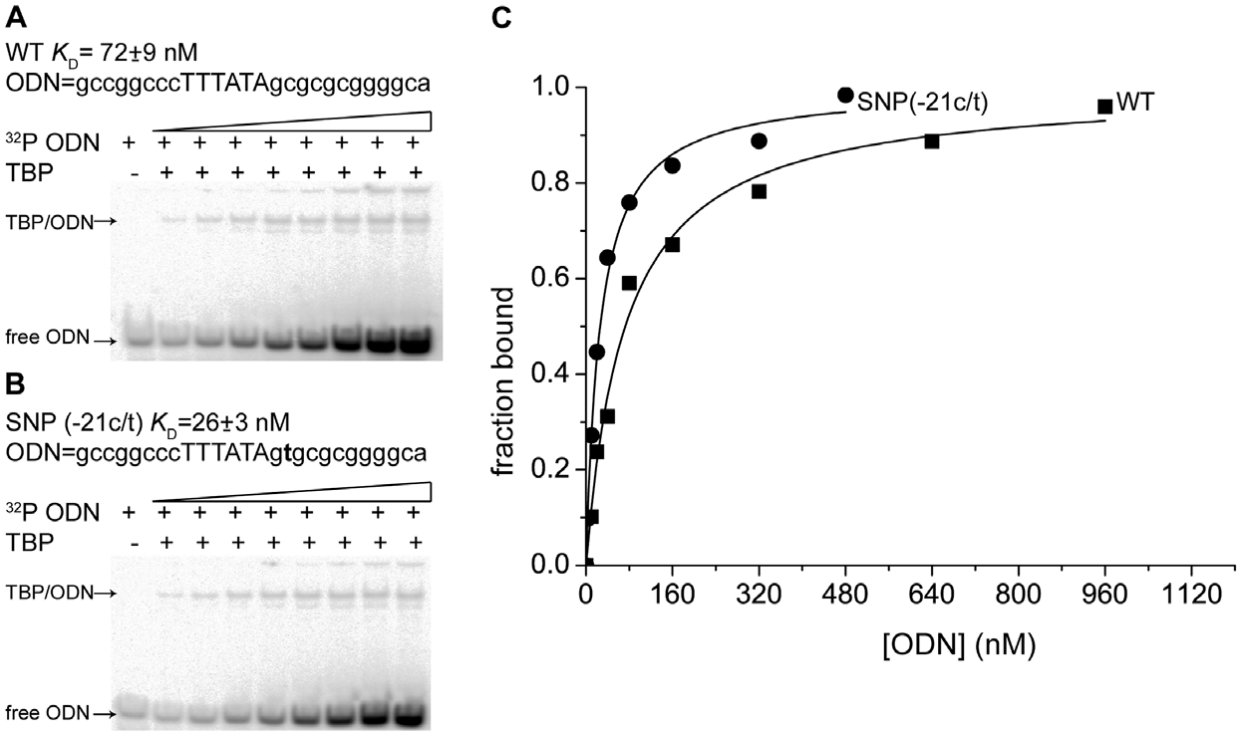
Experimental measurements of the equilibrium *K*_D_ for hTBP/TATA complexes. The use of an electrophoretic mobility shift assay for measuring hTBP/TATA affinity: (**A**) a TATA-containing ODN in healthy people (the −21C allele); (**B**) an ODN with the TATA box containing the SNP associated with myocardial infarction and thrombophlebitis (the −21t allele); (**C**) TBP/TATA binding isotherms inferred from electrophoregrams using OriginPro 8.

Experiments on hTPB/ODN binding were run at 25°C in a buffer (20 mM HEPES-KOH (pH 7.6), 5 mM MgCl2, 70 mM KCl, 1 mM DTT, 100 µg/ml BSA, 0.01% NP-40, 5% glycerol) until equilibrated. The hTPB-ODN complexes were separated from the unbound ODN using a gel retardation assay (EMSA). Electrophoresis was performed using 5% PAGE in Tris-glycine buffer (PH 8.3) for 40 min at a temperature of 10°C and a field intensity of 25 V/cm. The gels were dried and exposed to an Imaging Screen-K (Kodak) for use with a Molecular Imager PharosFX Plus phosphorimager (Bio-Rad). The screen was scanned by the phosphorimager and the radioautographs were quantitated using Quantity One 4.5.0 software (Bio-Rad). The equilibrium K_D_ values for the hTPB-ODN complexes, which characterize the affinity of TPB for TATA boxes, were determined using OriginPro 8 (for an example, see Fig. 2).

### Prediction of hTBP/TATA-box Affinity

The affinity of hTPB to ODNs with and without SNP in their TATA boxes expressed as natural logarithms was calculated using the equation (equation obtained earlier [8] by determining *K*_D_ complexes TBP/TATA to eight independent human genes, one gene of rat and mouse one gene [7]) for TBP/TATA equilibrium binding in three successive steps: sliding [9] ↔ recognition [10] ↔ stabilization [11]:



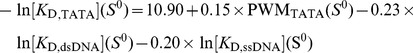
where 10.90 is the non-specific TPB/DNA affinity [4]; PWM_TATA_ is the highest score of Bucher's TATA-box weight matrix [10] from among all its 22 possible positions on the strand S^0^ and its complementary strand, each 26 bp long; ln[*K*_D_,dsDNA] is the mean of the regression of the affinity of TPB for the double-stranded DNA on the strand S^0^ that has the highest score of Bucher's TATA-box weight matrix (the sliding stage) [4], [9], [10]; ln[*K*_D_,ssDNA] is the half-sum of the regressions of TBP affinity for each strand on the sequences of each of these strands with the highest scores of Bucher's TATA-box weight matrix (the stabilization stage) [10], [50], [51]; 0.15, 0.23, 0.20 are the stoichiometric coefficients for three steps of TBP/TATA binding as calculated previously [8]. The difference (δ) between the affinity of hTPB for ODNs with and without SNPs in their TATA boxes expressed as natural logarithms: 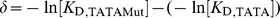


The coefficients of correlation (r) and their levels of significance (α) were calculated using the standard software program package STATISTICA [42].
